# In Situ Separation and Analysis of Lipids by Paper Spray Ionization Mass Spectrometry

**DOI:** 10.3390/molecules26010093

**Published:** 2020-12-28

**Authors:** Youngju Kal, Sangwon Cha

**Affiliations:** Department of Chemistry, Dongguk University, Seoul 04620, Korea; mg145@naver.com

**Keywords:** paper spray ionization, mass spectrometry, lipids, phosphatidylcholine, triacylglycerol

## Abstract

Paper spray ionization (PSI) is an extractive ambient ionization technique for mass spectrometry (MS), whereby a triangular paper tip serves as the sampling base and the electrospray tip. During PSI, analytes are extracted and transported to the edge of the paper tip by the applied spraying solvent. Analytes can be purified from a sample matrix and separated from each other by this transportation process. In this study, we investigated and utilized the analyte transportation process of PSI for the in situ separation and analysis of lipid mixtures. We found that differential transport of phosphatidylcholine (PC) and triacylglycerol (TAG), the two most abundant lipid classes in animals, occurred during PSI. We also found that the order in which these lipids moved strongly depended on how the spraying solvent was applied to the paper base. The more polar PC moved faster than the less polar TAG during PSI, when a polar solvent was slowly fed into a paper tip, whereas TAG was transported faster than PC when excess solvent was applied to the tip at once. In addition, we achieved a complete separation and detection of PC and TAG by slowly supplying a nonpolar solvent to a PSI tip.

## 1. Introduction

Ambient ionization methods for mass spectrometry (MS) have become immensely popular, as their convenience and raw material analysis capabilities are unapparelled [1,2]. The main advantage of ambient ionization lies in its ability to allow for direct sample analysis, with minimal sample preparation requirements [2]. Therefore, MS analysis employing an ambient ionization method can be utilized as a simple and rapid screening platform [3,4]. Since the development of desorption electrospray ionization (DESI) [5], a number of electrospray ionization (ESI)-based ambient ionization techniques have been developed. Among them, paper spray ionization (PSI) has become one of the most popular, due to its simplicity and low-cost [6,7,8].

In PSI, a planar triangular-shaped paper is used as an electrospray tip, a sampling base, and a separation medium. A liquid-phase sample is loaded onto the paper tip and dried; subsequently, a high voltage and spraying solvent are applied to paper [6]. These steps result in analyte transportation through the paper tip and ion generation at the edge of the paper tip via ESI-like processes, after which the generated analyte ions are directed to the mass spectrometer [6,9].

In addition to its simplicity and cost-effectiveness, PSI MS has displayed unique advantages. Firstly, due to the porous nature of paper, PSI does not give rise to clogs, even when crude or whole-blood samples are directly loaded on a paper tip [7,10]. For example, herbicides in an environmental sample matrix that could be directly analyzed by PSI MS, without the need to implement any filtering or sample cleanup procedures, even when the sample contained particulate matter [11]. Secondly, in contrast to conventional ESI, PSI has shown to be compatible with the use of nonpolar solvents like hexane, and it thus allows ionization of low-polarity and nonpolar saturated hydrocarbons and aromatic compounds [12,13]. Thirdly, since the surface characteristics of a paper tip can be readily engineered via simple chemical derivatization procedures like silanization [14] or by coating it with functionalized nanomaterials [15,16,17], a wide range of analytes can be analyzed by PSI MS with high selectivity and sensitivity. For example, 2,4,6-trinitrotoluene (TNT) could be sensitively detected by PSI with a platinum nanocomposite-embedded paper tip and a sodium borohydride-containing spraying solvent by converting TNT in situ to the more easily ionizable 2,4,6-triaminotoluene [18].

Besides the unique characteristics of PSI mentioned above, in the present study we specifically focused on utilizing the analyte transportation process of PSI. Previous studies on the analyte dissolution and transportation taking place during PSI indicate capillary action, electrophoresis, and bulk solution movement to be essential analyte transportation processes [8,19]. Specifically, capillary action is the main mechanism of analyte transport when a solvent is fed slowly to the paper tip using a syringe pump (wicking mode) [8,19]. For example, when a mixture of two dyes, methylene blue and methyl violet 2B, was analyzed by PSI MS in wicking mode, methyl violet 2B, which is characterized by weaker affinity for the paper substrate than methylene blue, was predominantly detected in the beginning of data acquisition, whereas methylene blue was dominant at the end of it [8]. However, bulk solution movement is favored when large amounts of the solvent are dispensed onto the paper surface at once (dumping mode), as the surplus volume of liquid unabsorbed by the paper medium readily forms a liquid film. When the same dye mixture mentioned above was analyzed, employing the dumping mode, the extent of separation between the two dyes was diminished [8].

In this study, we investigated the analyte transportation process of PSI in the case of a mixture of two compounds belonging to the two most abundant lipid families found in animal lipid extracts: phosphatidylcholine (PC) and triacylglycerol (TAG). Notably, both PC and TAG have a glycerol moiety. In PC, two fatty acids and one phosphocholine are covalently linked to the mentioned glycerol moiety, whereas, in the case of TAG, three fatty acids are attached to a glycerol moiety [20,21]. In fact, the presence of the phosphocholine group renders PC generally more polar than TAG. Our results indicated that PSI performed using methanol (MeOH), the most widely used polar spraying solvent in PSI, could induce differential transports of PC and TAG via both the wicking and dumping modes. Notably, this phenomenon was confirmed in the analysis of a more complex sample, a beef lipid extract. Furthermore, we successfully demonstrated in situ normal-phase-chromatography-like separation of these lipids by performing PSI MS with a nonpolar solvent in wicking mode.

## 2. Results and Discussion

### 2.1. Differential Transport of Lipids in PSI with a Polar Solvent

**Figure 1:**
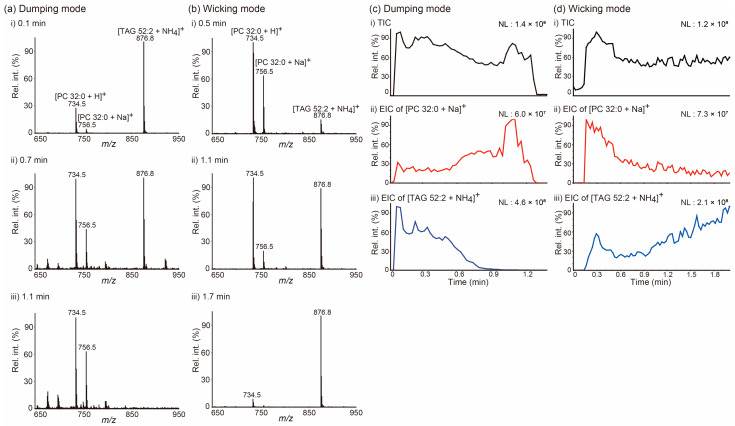
(**a**,**b**) Averaged paper spray ionization (PSI) mass spectra of a mixture of 1,2-dipalmitoyl-*rac*-glycero-3-phosphocholine (PC 32:0) and 1,3-dioleyl-2-palmitoyl-glycerol (TAG 52:2) at the indicated acquisition times. (**c**,**d**) (i) Total ion chronograms (TICs) of PSI mass spectrometry analysis of a mixture of PC 32:0 and TAG 52:2 and extracted ion chronograms (EICs) for (ii) the [PC 32:0 + Na]^+^ ion at *m/z* 756.5 and (iii) the [TAG 52:2 + NH_4_]^+^ ion at *m/z* 876.8. The PSI solvent (5 mM NH_4_Ac in MeOH) was applied either (**a**,**c**) in the dumping or (**b**,**d**) wicking modes. NL denotes the normalized level. MeOH: methanol; NH_4_Ac: ammonium acetate.

We selected two different lipids, a PC and a TAG, which are the most abundant lipid species found in animal lipid extracts, with the goal of exploring the issue of how lipids move on a paper tip during PSI MS in a manner dependent on the solvent application method. PSI mass spectra in Figure 1a,b were obtained from a mixture of 1,2-dipalmitoyl-*rac*-glycero-3-phosphocholine (PC 32:0) and 1,3-dioleyl-2-palmitoyl-glycerol (TAG 52:2) for the indicated acquisition time, when 5 mM ammonium acetate (NH_4_Ac) in MeOH was applied onto a tip employing the dumping (Figure 1a) and wicking (Figure 1b) modes. When the solvent was applied by the dumping mode, TAG 52:2 was the dominant species at the beginning of the acquisition (0.1 min), both PC 32:0 and TAG 52:2 were detected at comparable intensities in the middle of it (0.7 min), and PC 32:0 became the dominant species at the end of PSI (1.1 min). However, PSI MS analysis was carried out implementing the wicking mode that exhibited an almost opposite trend: PC 32:0 was the dominant species at the beginning (0.5 min), both PC and TAG were detected at comparable intensities in the middle (1.1 min), and TAG 52:2 was the dominant species at the end of the acquisition (1.7 min). These trends were further clearly recognized in the extracted ion chronograms (EICs) of the [PC 32:0 + Na]^+^ and [TAG 52:2 + NH_4_]^+^ ions (Figure 1c,d). Notably, lipids were detected as soon as PSI started when the solvent was applied in the dumping mode, whereas when solvent application was conducted in wicking mode, some time was necessary for lipids to be transported and detected, due to the slow solvent feeding rate.

**Figure 2:**
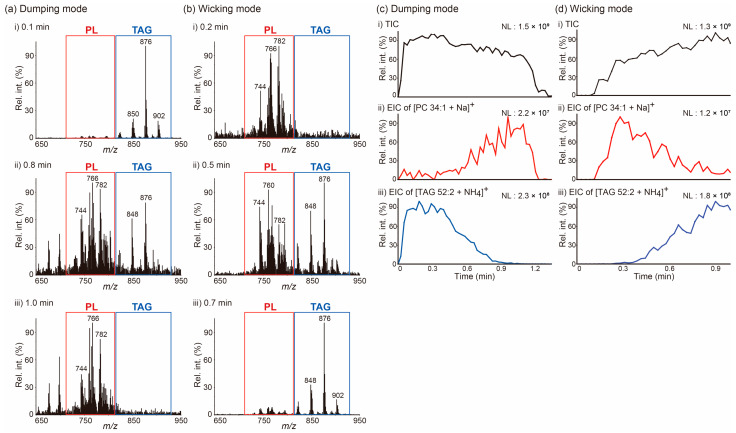
(**a**,**b**) Averaged paper spray ionization (PSI) mass spectra of a beef lipid extract recorded at the indicated acquisition times. (**c**,**d**) (i) Total ion chronograms (TICs) of PSI mass spectrometry analysis of a beef lipid extract and extracted ion chronograms (EICs) for (ii) the [PC 34:1 + Na]^+^ ion at *m/z* 782 and (iii) the [TAG 52:2 + NH_4_]^+^ ion at *m/z* 876. A PSI solvent (5 mM NH_4_Ac in MeOH) was applied either (**a**,**c**) via the dumping or (**b**,**d**) wicking modes. NL denotes the normalized level. MeOH: methanol; NH_4_Ac: ammonium acetate; PC 34:1: phosphatidylcholine 34:1; TAG 52:2: 1,3-dioleyl-2-palmitoyl-glycerol.

After observing the differential movements of lipids present in a simple mixture of two lipids as a result of changes in the solvent application method, we applied the same approach to the analysis of a more complex lipid mixture, a beef lipid extract. The results from these experiments are reported in Figure 2. Since the results of previous analyses performed on a beef lipid extract by matrix-assisted laser desorption/ionization MS indicated that phospholipids (PLs) were mainly detected in the 720–810 *m/z* region, whereas signals due to TAGs mainly appeared in the 810–920 *m/z* region [22,23], we could easily determine whether PLs or TAGs were the main species detected at a certain time point of the acquisition (Table 1). As can be evinced from the data in Figure 2a,b, the PSI mass spectra of a beef lipid extract displayed similar patterns to those obtained from a mixture of PC 32:0 and TAG 52:2. In the dumping mode, TAGs were the dominant species detected in the beginning (0.1 min), both PLs and TAGs were detected in the middle (0.8 min), and PLs were the dominant species detected at the end (1.0 min) of the acquisition. By contrast, the results of the analysis were performed applying the wicking mode showed an opposite order of analyte detection. The EICs of [PC 34:1 + Na]^+^ at *m/z* 782 and [TAG 52:2 + NH_4_]^+^ at *m/z* 876 allow to visualize this trend more clearly (Figure 2c,d). These results suggest that differential movements of lipids on a paper tip during the PSI process can occur when a polar solvent is utilized and that the method of solvent application is the primary determinant for how analytes are differentially transported during PSI. Understanding this phenomenon could be beneficial in the analysis of complex lipid mixtures via PSI MS, given that these differential transports of analytes may reduce spectral complexity, thereby reducing ion suppression and matrix effects. Furthermore, the separation of lipid classes that takes place during PSI can provide additional time to perform data-dependent MS/MS acquisitions aimed at the identification of lipid species.

**Figure 3:**
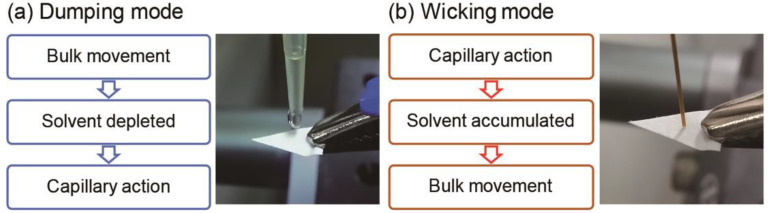
Analyte transportation processes taking place in paper spray ionization mass spectrometry, depending on the method of solvent application. (**a**) In the dumping mode, analyte transportation via bulk solution movement takes place during the early stages. As PSI proceeds, the spraying solvent becomes depleted, and capillary action becomes the dominant mode of analyte transport. (**b**) In the wicking mode, analytes are transported via capillary action during the early stages. As PSI proceeds, the spraying solvent becomes accumulated, and analyte transportation via bulk solution movement is initiated.

Differential transportation processes during PSI, induced by implementing the dumping and wicking modes, are illustrated in Figure 3, and provide possible explanations for the observed phenomena. In the dumping mode, since an excess volume of spraying solvent is dispensed onto the paper base at once, a liquid film initially forms on the paper tip and analyte transportation via bulk solution movement takes place during the early stages of the PSI process. In this scenario, the less polar TAG, which interacts with the paper base less strongly than the more polar PC, is the dominant species being transported. As PSI proceeds, the spraying solvent becomes depleted, and the liquid film may disappear. Therefore, capillary action becomes the dominant mode of analyte transport, and PC, which probably moves through the paper substrate more favorably than TAG, is mainly detected at the end of solvent spraying. By contrast, in the wicking mode, the analyte transportation sequence is opposite to that taking place in the dumping mode. In the beginning of the PSI process, a paper base is slowly dampened via capillary action, but the solvent volume is insufficient for a liquid film to form on the paper base. In this situation, analytes are transported via capillary action and PC is transported first. However, after delivering enough solvent volume to form a liquid film, analyte transportation via bulk solution movement is initiated, and the less strongly interacting TAG species are favored to be transported and detected.

**Figure 4:**
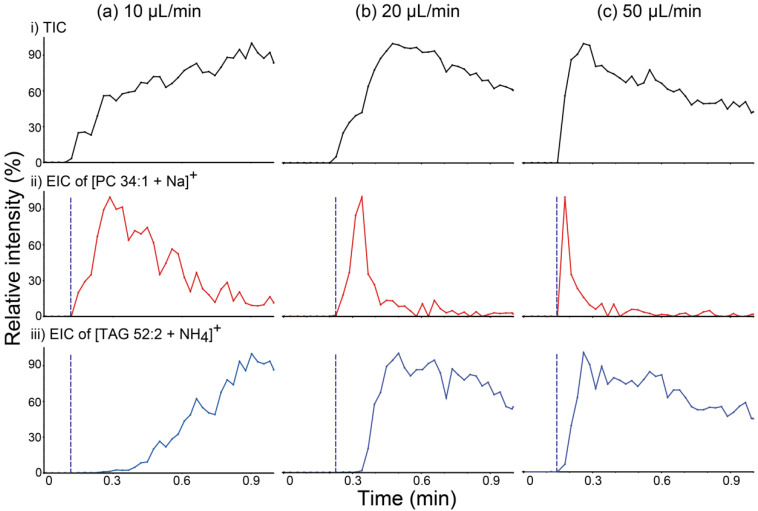
(**a**–**c**) (i) Total ion chronograms (TICs) of paper spray ionization mass spectrometry (PSI MS) analysis of a beef lipid extract and extracted ion chronograms (EICs) for (ii) the [PC 34:1 + Na]^+^ ion at *m/z* 782.8 and (iii) the [TAG 52:2 + NH_4_]^+^ ion at *m/z* 876.8. PSI MS analyses were performed implementing the wicking mode for solvent application at various solvent feeding rates: (**a**) 10, (**b**) 20, and (**c**) 50 µL/min. Blue dashed lines were inserted into EICs for comparing the detection times of lipids belonging to the phosphatidylcholine (PC) and triacylglycerol (TAG) classes. PC 34:1: phosphatidylcholine 34:1; TAG 52:2: 1,3-dioleyl-2-palmitoyl-glycerol.

In order to confirm the relationship between the liquid film formation and the characteristics of analyte transport, PSI MS analysis of a beef lipid extract was performed in wicking mode with various solvent feeding rates, and the resulting total ion chronograms (TICs) and EICs of [PC 34:1 + Na]^+^ at *m/z* 782 and [TAG 52:2 + NH_4_]^+^ at *m/z* 876 are shown in Figure 4. If TAG had mainly been transported via bulk solution movement, as speculated above, the rate of transport of this species would have increased as the solvent feed rate increased, which would result in the shortening of the time required for liquid film formation. As expected, the difference in detection time between PC and TAG species decreased as the solvent feed rate increased. These findings suggest that the presence of a surface liquid film is one of the critical factors determining the differential movement of PC versus TAG species.

### 2.2. Differential Elution Behavior of Lipids in PSI Performed with a Nonpolar Solvent

Given that PSI has a much wider solvent compatibility than conventional ESI [12,13], PSI MS analyses were also performed on a lipid mixture using nonpolar solvents like hexane. In such experiments, lipids may be separated according to their polarities, because differential lipid retentions may occur between a polar paper tip and a nonpolar solvent. In other words, PSI processes conducted with a nonpolar solvent could induce normal-phase-chromatography-like separations of lipids.

**Figure 5:**
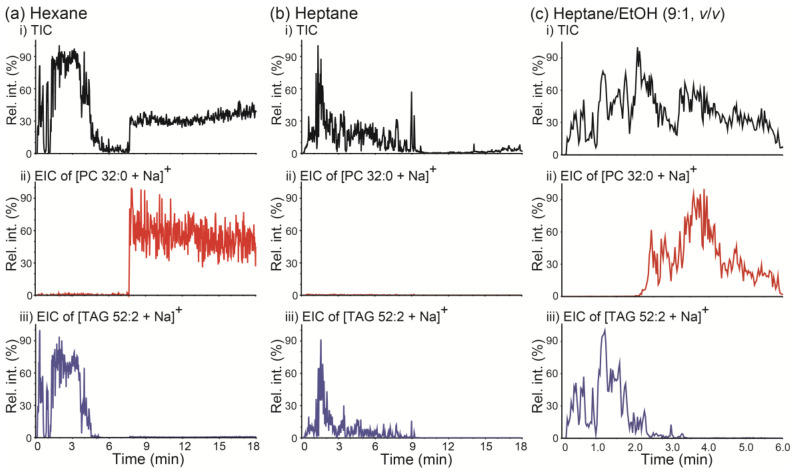
(**a**–**c**) (i) Total ion chronograms (TICs) of paper spray ionization mass spectrometry (PSI MS) analysis of a mixture of PC 32:0 and TAG 52:2 and extracted ion chronograms (EICs) for (ii) the [PC 32:0 + Na]^+^ ion at *m/z* 756.5 and (iii) the [TAG 52:2 + Na]^+^ ion at *m/z* 881.8. PSI MS was performed by wicking mode with (**a**) hexane, (**b**) heptane, and (**c**) heptane/ethanol (EtOH) (9:1, *v/v*) as spraying solvents. PC 32:0: 1,2-dipalmitoyl-*rac*-glycero-3-phosphocholine; TAG 52:2: 1,3-dioleyl-2-palmitoyl-glycerol.

PSI MS analysis was performed on a mixture of PC 32:0 and TAG 52:2 using hexane as the solvent and implementing the wicking mode. The resulting TIC and EICs of [PC 32:0 + Na]^+^ (*m/z* 756.5) and [TAG 52:2 + Na]^+^ (*m*/*z* 881.8) are displayed in Figure 5a. Two clearly separate TIC regions were observed (i.e., 0–5 min and 8–18 min), and the dominant lipids detected in each region were found to be TAG 52:2 (0–5 min) and PC 32:0 (8–18 min), respectively. When hexane was used as the spraying solvent, such differential retentions of TAG and PC were reasonable, because under normal-phase-chromatography-like conditions the less polar TAG is expected to be less retained on a polar paper tip than the more polar PC. When heptane was employed as a solvent instead of hexane, only TAG 52:2 was detected, while PC 32:0 was retained on the paper base for at least 18 min (Figure 5b). These results suggest that normal-phase-chromatography-like separation of lipids is possible by PSI MS using a nonpolar solvent; moreover, implementing PSI MS using a nonpolar solvent can be beneficial in the analysis of complex lipid mixtures. Notably, the degree of differential retention of PC versus TAG could be controlled by modifying solvent polarity. When a polar organic solvent like ethanol (EtOH) was added to heptane (9:1 heptane/EtOH, *v*/*v*), both PC 32:0 and TAG 52:2 could be detected within 6 min, and a reasonable separation between these two lipids was observed.

## 3. Materials and Methods

### 3.1. Reagents and Materials

Ammonium acetate (NH_4_Ac), 1,2-dipalmitoyl-*rac*-glycero-3-phosphocholine (PC 32:0), 1,3-dioleyl-2-palmitoyl-glycerol (TAG 52:2), methanol (MeOH), ethanol (EtOH), isopropyl alcohol (IPA), chloroform, hexane, and heptane were obtained from Sigma-Aldrich (St. Louis, MO, USA). The eye-of-round ground beef was purchased from a local grocery store. Grade 1 filter paper was purchased from Whatman (Maidstone, England).

### 3.2. Preparation of a Standard Lipid Mixture and a Beef Lipid Extract

A standard lipid mixture of PC 32:0 and TAG 52:2 was prepared in chloroform, at a final concentration of 1 mg/mL of each lipid. Beef lipids were extracted from the eye-of-round ground beef (0.25 g) by adding to the beef sample 5 mL of an extraction solvent [hexane/IPA (3:2, *v/v*)] and subjecting the mixture thus obtained to a 10-min vortexing session. Then, the beef residue was spun down, and the supernatant was collected.

### 3.3. PSI MS

For PSI MS analysis, 1 μL of sample solution was loaded onto the middle of the triangular paper tip (base: 5 mm; height: 10 mm) prepared with a Grade 1 filter paper. After a sample was dried, the sample-loaded paper tip was connected to a high-voltage power supply with an alligator clip, before being placed 5 mm in front of a mass spectrometer inlet. Spraying solvent and high voltage were then applied to the paper tip for MS data acquisition. The application of the spraying solvent was conducted in one of two ways: implementing the dumping or wicking modes. In the dumping mode, 20 μL of spraying solvent were applied on the paper tip at once using a micropipette. By contrast, the wicking mode involved the spraying solvent being delivered through a syringe pump via fused silica capillary (360 μm OD and 100 μm ID, Polymicro Technologies LLC, Phoenix, AZ, USA) with solvent feed rates being held at 10 and 20 μL/min for polar and nonpolar organic spraying solvents, respectively, unless otherwise noted. In the case whereby MeOH was used as the spraying solvent, NH_4_Ac was added to it to a final concentration of 5 mM, so as to enhance the intensity of the signals due to TAGs, as well as to reduce spectral complexity. All mass spectra were recorded with the Thermo Finnigan LCQ Deca XP MAX quadrupole ion trap mass spectrometer (Thermo Scientific Inc., San Jose, CA, USA). The spraying voltage used for PSI MS was +4.5 kV, while the capillary voltage and temperature were set to 35 V and 250 °C, respectively.

## 4. Conclusions

In this study, the characteristics of differential analyte transport during PSI as determined by the identity of the solvent utilized were explored by performing PSI MS analyses on simple and complex lipid mixtures. When a polar spraying solvent was employed, the amount of solvent loaded onto a paper tip at a given time point determined the degree of liquid film formation as well as the favored transportation process. When a nonpolar spraying solvent was utilized, the polar paper tip and the nonpolar solvent acted like the stationary and mobile phases, respectively, of a normal-phase chromatography experiment, and PSI MS afforded the complete separation and detection of a PC lipid and a TAG lipid in situ.

## Figures and Tables

**Table 1:** Major lipid ions detected in a beef lipid extract by paper spray ionization mass spectrometry.

*m/z* ^a^	Possible Ion Assignment ^b^	*m/z*	Possible Ion Assignment
744	[PE 36:2 + H]^+ c^	808	[PC 36:2 + Na]^+^
756	[PC 32:0 + Na]^+^	848	[TAG 50:2 + NH_4_]^+^
760	[PC 34:1 + H]^+^	876	[TAG 52:2 + NH_4_]^+^
766	[PE 36:2 + Na]^+^	902	[TAG 54:3 + NH_4_]^+^
782	[PC 34:1 + Na]^+^	904	[TAG 54:2 + NH_4_]^+^

^a^ Indicated *m/z* values are nominal *m/z* values. ^b^ Peak assignments were based on previously reported identifications [22,23]. ^c^ PE denotes phosphatidylethanolamine.

## Data Availability

The data within the article are available from the corresponding author upon request.
